# The Effect of Modified Tai Chi Exercises on the Physical Function and Quality of Life in Elderly Women With Knee Osteoarthritis

**DOI:** 10.3389/fnagi.2022.860762

**Published:** 2022-05-26

**Authors:** Jiulong Song, Lijun Wei, Kai Cheng, Qiang Lin, Peng Xia, Xinwei Wang, Xiaoju Wang, Ting Yang, Baoyi Chen, Aimei Ding, Mingyi Sun, Anliang Chen, Xueping Li

**Affiliations:** ^1^Department of Sports and Health, Nanjing Sport Institute, Nanjing, China; ^2^Department of Rehabilitation Medicine, Nanjing First Hospital, Nanjing Medical University, Nanjing, China; ^3^Maigaoqiao Community Health Service Center, Nanjing, China; ^4^Department of Tourism and Social Management, Nanjing Xiaozhuang University, Nanjing, China

**Keywords:** modified Tai Chi, elderly women, knee osteoarthritis, physical function, quality of life

## Abstract

**Background:**

Knee osteoarthritis (KOA) is the leading cause of pain and stiffness, affecting older adults’ physical function and quality of life. As a form of mind-body exercise, Tai Chi has been recommended as an exercise prescription for KOA patients. This study examined the effects and continuation of modified Tai Chi exercises on physical function and quality of life in elderly women with KOA.

**Methods:**

We conducted a single-blind, randomized controlled trial (RCT) on 40 older women with KOA. The participants were randomized to a 12 weeks Tai Chi or control group. The Tai Chi group attended a kind of modified Tai Chi training sessions three times per week; the control group attended wellness education sessions once a week. The primary outcome was the Western Ontario and McMaster University Osteoarthritis Index (WOMAC). Secondary outcomes were the Berg Balance Scale (BBS), Timed Up and Go (TUG), Short-Form 36 (SF-36), Pittsburgh Sleep Quality of Index (PSQI), Self-rating Anxiety Scale (SAS), and Self-rating Depression Scale (SDS).

**Results:**

After the 12-weeks the Tai Chi group showed significan improvement in the WOMAC pain (mean difference, −5.09 points, *p* = 0.001), WOMAC stiffness (mean difference, −3.60 points, *p* = 0.002), WOMAC physical function (mean difference, −11.21 points, *p* = 0.001) compared to the control group. In addition, the Tai Chi group had also significant improvement in the BBS (mean difference, 1.70 points, *p* = 0.008), TUG (mean difference, −0.52s, *p* = 0.001), SF-36PCS (mean difference, 7.60 points, *p* = 0.001), MCS (mean difference, 7.30 points, *p* = 0.001), PSQI (mean difference, −3.71 points, *p* = 0.001), SDS (mean difference, −5.37 points, *p* = 0.025) and SAS (mean difference, −5.06 points, *p* = 0.002).

**Conclusion:**

The modified Tai Chi exercises are an effective treatment for improved physical function and quality of life in elderly women with KOA.

**Clinical Trial Registration:**

The trial was registered in Chinese Clinical Trial Registry (ChiCTR2000040721), http://www.chictr.org.cn/edit.aspx?pid=65419&htm=4.

## Introduction

Knee osteoarthritis (KOA) is one of the common chronic joint diseases in the elderly population (Sharma, 2021), resulting in pain, physical functional limitations, and low quality of life (Lee et al., 2018; Hawker, 2019). Epidemiological studies show that 8.1% of the elderly (age ≥60) in China suffer from symptomatic KOA. The prevalence of KOA in women is 10.3%, higher than that of 5.7% in men (Tang et al., 2016; Vinaa and Kwoha, 2018); KOA affects women more frequently than men and has a greater impact on women’s physical health (Yue et al., 2020). Patients with KOA commonly suffer from decreased physical function and quality of life due to joint pain and limited physical activity (Rosemann et al., 2007). In addition, increased pain and loss of physical function in KOA patients also progressively worsen their quality of life (Dominick et al., 2004). A cross-sectional study showed that KOA patients had lower scores in physical activity, mental health, and sleep disturbances than healthy people (Cook et al., 2007).

At present, conventional treatment methods for KOA include pharmacological interventions, physical therapy, and surgical treatment (Hochberg et al., 1995; Hermann et al., 2018; Sari et al., 2019). Exercise therapy is an essential part of KOA management; it is beneficial in reducing joint pain, improving functional impairment, and preventing joint mobility limitations (Juhl et al., 2014). Indeed, several studies have shown that exercise can relieve the symptoms of KOA by increasing muscle strength, improving neuromotor control, and optimizing joint range of motion (Woo et al., 2007; Zhu et al., 2016). A systematic review indicated that exercise could significantly reduce pain and improve physical function in KOA (Fransen et al., 2015). Currently, there is not enough evidence to recommend that one kind of exercise is better than the other, but patients can choose according to their preferences and circumstances, both of which determine the enthusiasm of patients to participate in the exercise (Kolasinski et al., 2020).

Tai Chi is a traditional Chinese mind-body exercise that combines meditation, breathing, and movement and effectively relieves pain, prevents falls, and improves anxiety and depression (Li et al., 2001; Lan et al., 2002). A systematic review found short-term improvement in pain, physical function, and stiffness in patients with KOA practicing Tai Chi (Lauche et al., 2013). In a previous randomized, single-blind trial comparing Tai Chi and physical therapy, Tai Chi exerted an effect similar to physical therapy in treating KOA; it gave rise to a more significant improvement in depression and quality of life (Wang et al., 2016). In 2019, the American College of Rheumatology recommended Tai Chi intervention as an exercise prescription for KOA (Kolasinski et al., 2020).

Although the evidence shows that Tai Chi is safe (Shengelia et al., 2013), some difficult movements in traditional Tai Chi exercises may exert excessive loads on the lower limb joints in KOA patients with physical dysfunction. However, previous studies have paid little attention to this issue. Therefore, our research was designed for this characteristic of KOA patients. Firstly, we assessed each participant’s balance; secondly, each participant’s range of motion and pain were assessed using a joint range of motion and pain assessments. Finally, we personalize it based on participants’ characteristics and expert opinions. This study aimed to examine the effect of a 12-week modified Tai Chi exercise on the physical function and quality of life with knee OA and the continuation of subsequent treatment effects. We hypothesized that modified Tai Chi exercises could improve physical function and quality of life in patients with KOA.

## Materials and Methods

### Trial Design

This parallel, single-blind, randomized controlled trial compared the effect of Tai Chi exercises and wellness education on the physical function and quality of life in older women with KOA. The participants were randomly assigned to the Tai Chi or control groups using a computer-generated random number. This study was approved by the Nanjing Sport Institute Ethics Committee, and all the patients submitted informed consent to participate in the study.

### Participants

The participants were recruited in a community free clinic through WeChat public account promotion, advertisements, and recommendations from a community chronic disease management center. For interested respondents, we conducted a simple screening; subjects who met the KOA standard underwent a radiographic examination after signing the informed consent form to further ensure that they met the requirements of KOA.

Primary inclusion criteria for participants were: (1) women 60–75 years of age; (2) clinical radiographic diagnosis of KOA, and reported a 1–2 year course of disease; (3) pain symptoms for at least 6 months; (4) Kallgren-Lawrence (K/L) grading scale (0–3) for KOA; radiographs showing the knee space narrowing, with no large loose bodies in the joints; (5) no history of Tai Chi exercises; and (6) having signed the informed consent form.

Exclusion criteria for participants were: (1) severe cardiac, hepatic, and renal diseases preventing relaxed exercises; (2) knee pain or deformity or inconveniences in the spine, hips, ankles, and feet; (3) other diseases such as knee tumors, tuberculosis, infections, rheumatoid conditions, etc.; (4) individuals having undergone hip, knee, and ankle surgeries within 6 months before treatment; (5) having taken medicines for knee conditions within 3 months before treatment and those treated by injections; and (6) subjects with consciousness disorders or inability to cooperate.

### Interventions

#### The Tai Chi Group

The Tai Chi group participated in a 60-min session and repeated it three times a week for 12 weeks. Each session is conducted in the group within the community center, with a 10-min warm-up, 40-min Tai Chi practice, and cooling down for 10 min instruction by a professional Tai Chi master. The Tai Chi programs were adapted from the classical Yang style 24-form Tai Chi exercises. According to the characteristics of the subjects and the opinions of experts, to reduce sustained weight-bearing time of the knee joint and excessive knee flexion movements among traditional 24-form that included eight forms: (1) “Part the wild horse’s mane;” (2) “Brush the knee and twist steps;” (3) “Step back to repulse monkey;” (4) “Grasp sparrow’s tail on both sides;” (5) “Wave hands like clouds;” (6) “Golden pheasant stands with one leg;” (7) “Jade lady weaves shuttles;” and (8) “Apparent close-up.” An emphasis was placed on the transfer of the body’s center of gravity and multi-segment movement coordination of the trunk, incorporating regular breathing as part of the exercise and integrating it into the practice. After the 12 weeks, we encouraged patients to continue practicing Tai Chi and re-evaluated the participants in 3- and 6-months respectively.

#### The Control Group

The control group participated in the 12-week program (60-min per session, once per week). Each session consisted of 45-min health education, including disease prevention, health management, self-care, etc. Concerning the 15-min home-based self-exercise guidance, its content was light intensity resistance exercise and stretching of the lower limbs. The participants were encouraged to practice at home at least twice per week and maintain their original lifestyle habits. During the 12-week intervention period, the attendance was recorded as a check-in card method, and participants with an attendance rate of more than 80% received a gift after the experiment to assess the effectiveness of the intervention.

### Outcomes

The outcome measures were assessed at baseline and 12-week, 3-month, and 6-month follow-up, including the subject’s demographic and clinical characteristics and primary and secondary outcome measures. All the research measures were professional to avoid subjective errors; the same assessors conducted each test.

#### Primary Outcome Measures

The primary outcome measures were the change in the Western Ontario and McMaster University Osteoarthritis Index (WOMAC) score at baseline and after treatment of 12-week. The WOMAC is a self-report questionnaire for KOA to assess pain, stiffness, and physical function. Its reliability was satisfactory, with ICCs of 0.86, 0.68, and 0.89 (Salaffi et al., 2003). It includes five items for pain (score range: 0–50), two items for stiffness (score range: 0–20), and 17 items for physical function (score range: 0–170).

#### Secondary Outcome Measures

Secondary outcome measures included physical function and quality of life. The Berg Balance Scale (BBS) and Time Up and Go test (TUG) was assessed as physical function. BBS was widely used in clinical testing of human static and dynamic balance ability; the reliability was high (0.98). It consists of 14 items; each item is scored from 0 (cannot be completed) to 4 points (normal completion) with a total score range of 0–56, where a higher score indicates a better balance (Downs et al., 2013). TUG is a quick and quantitative method to assess functional walking ability in which the participant was asked to rise from a 45-cm chair, walk 3 meters, turn around, walk back to the chair, and then sit (Podsiadlo and Richardson, 1991).

Health-related quality of life assessment was conducted using the MOS item Short-Form 36 (SF-36), Pittsburgh Sleep Quality Index (PSQI), Self-rating Anxiety Scale (SAS), and Self-rating Depression Scale (SAS). SF-36 is currently used widely to assess the general population’s quality of life and evaluate clinical trial effects. It includes eight domains, internal consistency of each of the eight subscales was excellent (Cronbach’s coefficient alpha range 0.64–0.93). Our study used Physical components summary (PCS) and Mental components summary (MCS) as evaluation indicators (Laeson, 1997). PSQI is a self-rated questionnaire to assess sleep quality and disturbances; its reliability was 0.87. It contains seven components, ranging from 0 to 21; the higher scores indicate poorer sleep quality (Buysse et al., 1989). SAS and SDS were used to assess anxiety and depression symptoms. It includes 20 items, with a 1–4 scale; a higher score indicates more severe symptoms. Cronbach’s alpha for the SAS and SDS in this study was 0.83 and 0.73 (Dunstan et al., 2017; Dunstan and Scott, 2020).

### Sample Size

According to G-power software, a two-sided *t*-test was used to estimate the sample size. When the effect size was 1.18, *a* = 0.05, and the power was 0.9, considering the 20% attrition rate, the total sample size was calculated to be at least 40 people.

### Randomization and Blinding

Randomization included computer-generated numbers for random grouping, set random number seeding, and random number generation function Rv. Random numbers were generated from 0 to 40, using a visual sub-box for regional segmentation, randomly assigning individuals to the Tai Chi and the control groups according to 1:1 proportion. A random number was placed in a sealed opaque envelope, which was opened for each subject separately after signing the informed consent form. The study assessors who performed the outcome measures were blinded to group assignment and the grouping researchers were not participate in the outcome measurement.

### Statistical Methods

All the analyses were based on the intention to treat. The demographic characteristics and baseline variables of the two groups were analyzed using the analysis of variance (ANOVA) for continuous variables and the chi-squared test for categorical variables. *Post-hoc* tests were used to compare factor means for significant models for the missing data. Linear mixed models were used to test the changes in various observation indicators between the two groups over time. For intra-group comparisons, changes in outcome variables between baseline, post-intervention, and follow-up time intervals were estimated from the mixed model, stratified by group. For inter-group comparisons, the difference in outcome variables between the two groups at each time interval was estimated by the mixed model and the interactions between groups and time. We report 95% confidence intervals (95% CI) and significance levels for the difference and trend tests for between-group comparisons. The data analyses were carried out using SPSS 25.0 (IBM SPSS Inc., Chicago, USA). The study test level was set at α = 0.05, and *p* < 0.05 was considered statistically significant.

## Results

**Figure 1:**
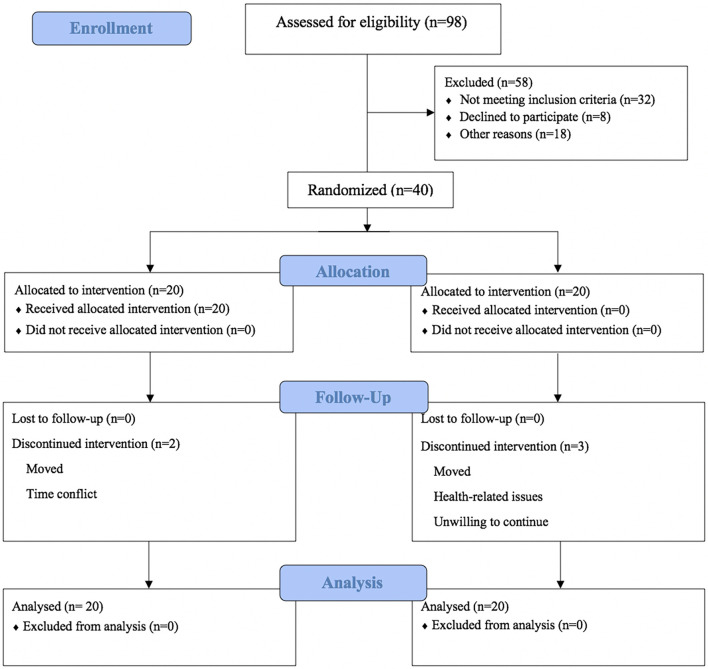
Study flow chart.

Ninety-eight individuals were recruited; 58 individuals were excluded for various reasons. The main reasons for exclusion were a lack of inclusion criteria (*n* = 32), no radiographic diagnostic evidence for KOA (*n* = 12), time conflict (*n* = 8), and other reasons (*n* = 6). Of these individuals, 40 were qualified and were randomly assigned to the Tai Chi (*n* = 20) and control (*n* = 20) groups (see Figure 1).

### Baseline Data

Table 1 presents baseline characteristics of participants, including age, BMI, course of the disease, K/L grade, comorbidities, and the outcome measures in the two groups. There were no significant differences between the Tai Chi and the control groups at baseline (*p* > 0.05).

**Table 1:** Baseline characteristics of participants*.

**Variable**	**Tai Chi group (n = 20)**	**Control group (n = 20)**	P**
Mean age, years	64.15 ± 8.56	64.15 ± 8.56	0.967
Body Mass Index, kg/m^2^	24.60 ± 5.64	24.37 ± 2.71	0.873
Course of disease, years	6.60 ± 2.01	5.60 ± 2.16	0.138
Radiograph score, n (%)			
K/L grade 1	7 (35)	7 (35)	
K/L grade 2	8 (40)	7 (35)	
K/L grade 3	5 (25)	6 (30)	0.924^χ^
Comorbidities, n (%)			
Hypertension	14 (43.8)	12 (33.3)	
Diabetes	10 (31.3)	15 (41.7)	
Osteoporosis	6 (18.8)	8 (22.2)	
COPD	2 (6.3)	1 (2.8)	0.672^χ^
WOMAC score			
Knee pain (0-50)^†^	21.22 ± 3.42	21.17 ± 3.81	0.970
Knee stiffness(0-20)^†^	10.27 ± 2.73	10.12 ± 3.27	0.876
Physical function (0-170)^†^	40.83 ± 5.23	40.23 ± 7.58	0.787
Balance function, Mean (SD)			
BBS (0-56)^‡^	52.27 ± 1.96	51.94 ± 2.07	0.625
TUG, seconds	10.13 ± 0.42	10.17 ± 0.51	0.769
Health-related quality of life			
SF-36 PCS (0-100)^‡^	58.33 ± 7.27	57.64 ± 6.15	0.766
SF-36 MCS(0-100)^‡^	57.38 ± 6.64	58.70 ± 5.04	0.515
Sleep quality			
PSQI (0-21)^†^	8.89 ± 4.28	9.47 ± 2.47	0.629
Anxiety and Depression			
SDS (20-80)^†^	42.55 ± 8.12	41.94 ± 5.84	0.800
SAS (20-80)^†^	39.94 ± 5.81	39.35 ± 3.80	0.726

*Values are shown as mean ± SD (Standard Deviation) unless otherwise indicated. **P values were calculated by *t*-test for continuous variables and by chi-square test for categorical variables. K/L, Kellgren-Lawrence scale; COPD, Chronic obstructive pulmonary disease; WOMAC, The Western Ontario and McMaster Universities Osteoarthritis Index; BBS, Berg balance scale; TUG, Time up go; SF-36, The MOS item short form health survey; MCS, Mental component summary; PCS, Physical component summary; PAQI, Pittsburgh sleep quality index; SAS, Self-rating Anxiety Scale; SDS, Self-rating Depression Scale. ^†^Lower scores indicated an improved state. ^‡^Higher scores indicated an improved state. ^χ^Chi-squared test.

Of the 40 randomized participants, 35 (87.5%) completed the follow-up assessment, including 18 (90%) in the Tai Chi group and 17 (85%) in the control group. Figure 1 presents the reasons for withdrawing or not completing post-intervention testing. In the intervention period, the attendance rate of the Tai Chi group was 88%, with 81% in the control group. Common reasons for absenteeism were time conflicts and health-related issues.

### Primary Outcomes

**Figure 2:**
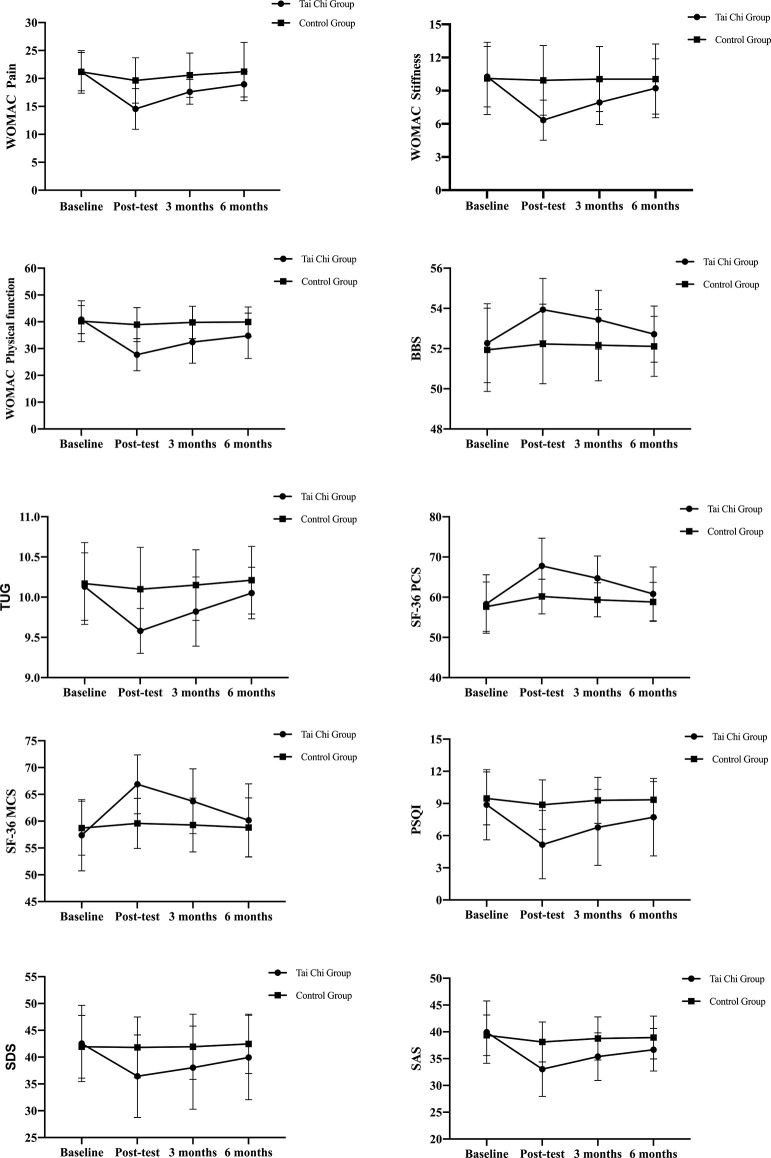
The changes in the indicators of the two groups at Baseline, 12 weeks, follow-up 3 months, and 6 months over time. The value is the mean ± standard deviation (SD); error bars show the 95% confidence interval. WOMAC, The Western Ontario and McMaster Universities Osteoarthritis Index; BBS, Berg balance scale; TUG, time up go; SF-36, The MOS item short-form health survey; MCS, Mental component summary; PCS, Physical component summary; PSQI, Pittsburgh sleep quality index; SAS, Self-rating Anxiety Scale; SDS, Self-rating Depression Scale.

At 12 weeks, there was a significant difference between the two groups in the WOMAC score. Compared with the baseline, the WOMAC score in the Tai Chi group was significantly improved while there was no significant improvement in the control group. Compared with the control group, the Tai Chi group showed significant improvements in WOMAC pain (*p* = 0.001), stiffness (*p* = 0.002), and physical function (*p* = 0.001). At the 3-month follow-up, there were still differences in the WOMAC scores between the two groups. However, at the 6-month follow-up, there was no significant difference between the two groups (Table 2, Figure 2).

**Table 2:** Primary and secondary outcomes.

**Variable**	Mean change from baseline*		Between-group difference (95%CI)**	
	**Tai Chi (*n* = 20)**	**Control (*n* = 20)**	**Tai Chi vs. Control**	***P* ^#^**
Primary outcome: WOMAC score				
Knee pain (0	#x02013;50)^†^				
Post-test	14.55 ± 3.64	19.64 ± 4.06	−5.09 (−7.74,−2.43)	0.001
Follow-up 3 months	17.61 ± 2.22	20.58 ± 3.95	−2.97 (−5.16,−0.78)	0.009
Follow-up 6 months	18.94 ± 2.26	21.23 ± 5.22	−2.91 (−5.03, 0.45)	0.099
Knee stiffness (0–20)^†^				
Post-test	6.33 ± 1.81	9.94 ± 3.15	−3.60 (−5.36,−1.85)	0.001
Follow-up 3 months	7.94 ± 2.01	10.05 ± 2.94	−2.11 (−3.84,−0.38)	0.018
Follow-up 6 months	9.22 ± 2.66	10.05 ± 3.17	−0.83 (−2.84,1.17)	0.404
Physical function (0–100)^†^				
Post-test	27.72 ± 6.01	38.94 ± 6.32	−11.21 (−15.46,−6.97)	0.001
Follow-up 3 months	32.44 ± 7.92	39.76 ± 6.04	−7.32 (−12.18,−2.45)	0.004
Follow-up 6 months	34.77 ± 8.46	39.94 ± 5.57	−5.16 (−10.12,−0.20)	0.042
Secondary outcomes				
BBS (0–56)^‡^				
Post-test	53.94 ± 1.55	52.23 ± 1.98	1.70 (0.48, 2.93)	0.008
Follow-up 3 months	53.44 ± 1.46	52.17 ± 1.77	1.26 (0.15, 2.38)	0.027
Follow-up 6 months	52.72 ± 1.40	52.11 ± 1.49	0.60 (−0.39,1.60)	0.226
TUG (s)				
Post-test	9.58 ± 0.28	10.10 ± 0.52	−0.52 (−0.81, −0.23)	0.001
Follow-up 3 months	9.82 ± 0.43	10.15 ± 0.44	−0.32 (−0.60,−0.05)	0.019
Follow-up 6 months	10.05 ± 0.32	10.21 ± 0.42	−0.15 (−0.41,−0.09)	0.218
SF-36 PCS (0–100)^‡^				
Post-test	67.77 ± 6.90	60.17 ± 4.31	7.60 (3.61,11.59)	0.001
Follow-up 3 months	64.72 ± 5.54	59.35 ± 4.22	5.36 (1.96, 8.77)	0.003
Follow-up 6 months	60.83 ± 6.69	58.82 ± 4.85	2.01 (−2.03, 6.05)	0.319
SF-36 MCS (0–100)^‡^				
Post-test	66.88 ± 5.49	59.58 ± 4.67	7.30 (3.78,10.82)	0.001
Follow-up 3 months	63.72 ± 6.03	59.29 ± 5.03	4.42 (0.59, 8.26)	0.025
Follow-up 6 months	60.16 ± 6.79	58.82 ± 5.51	1.34 (−2.92, 5.61)	0.527
PSQI (0–21)^†^				
Post-test	5.16 ± 3.18	8.88 ± 2.31	−3.71 (−5.64,−1.79)	0.001
Follow-up 3 months	6.77 ± 3.54	9.29 ± 2.14	−2.51 (−4.54,−0.48)	0.017
Follow-up 6 months	7.72 ± 3.61	9.35 ± 1.69	−1.63 (−3.59, 0.32)	0.100
SDS (20–80)^†^				
Post-test	36.44 ± 7.70	41.82 ± 5.67	−5.37 (−10.05,−0.70)	0.025
Follow-up 3 months	38.05 ± 7.75	41.94 ± 6.08	−3.88 (−8.70, 0.92)	0.110
Follow-up 6 months	39.94 ± 7.88	42.47 ± 5.54	−2.52 (−7.24, 2.18)	0.284
SAS (20–80)^†^				
Post-test	33.05 ± 5.09	38.11 ± 3.72	−5.06 (−8.14,−1.97)	0.002
Follow-up 3 months	35.38 ± 4.44	38.76 ± 4.02	−3.37 (−6.29,−0.45)	0.025
Follow-up 6 months	36.66 ± 3.98	38.94 ± 3.99	−2.27 (−5.01, 0.47)	0.101

*Values are the mean ± SD (Standard Deviation). **Values are shown as mean (95% confidence interval). WOMAC, The Western Ontario and McMaster Universities Osteoarthritis Index; BBS, Berg balance scale; TUG, Time up go; SF-36, The MOS item short form health survey; MCS, Mental component summary; PCS, Physical component summary; PAQI, Pittsburgh sleep quality index; SAS, Self-rating Anxiety Scale; SDS, Self-rating Depression Scale. ^#^*P*values were calculated with repeated-measures analysis of variance. ^†^Lower scores indicated an improved state. ^‡^Higher scores indicated an improved state.

### Secondary Outcomes

We compared the changes in balance function, quality of life, sleep status, anxiety, and depression in the two groups before and after 12 weeks. Compared with the control, the BBS score was higher in the Tai Chi group than the control group (*p* = 0.008), and the TUG time was significantly reduced by −0.52 s (*p* = 0.001). In SF-36, the Tai Chi group improved physiological function, which increased by 7.60 points (*p* = 0.001), with a 7.30-points increase in the mental health score (*p* = 0.001). The Tai Chi group had a more significant improvement in sleep quality (−3.71 points; *p* = 0.001). Between the SDS score and SAS score, SDS decreased by 5.37 points (*p* = 0.025); SAS decreased by 5.06 points (*p* = 0.002).

### Adverse Events

During the 12-week intervention, we used a standard adverse event report form to monitor adverse events during the study, including the start and end time, frequency, symptoms, signs, description of the extent of adverse events, and whether corresponding measures were taken. Due to the nature of the experiment, thigh muscle soreness and other symptoms occurred after Tai Chi exercise, mainly caused by the accumulation of lactic acid, which was normal. Therefore, it was not considered an adverse event.

## Discussion

This study explored the effects of the modified Tai Chi exercise on the physical function and quality of life in older women with KOA. The results showed that 12-week Tai Chi exercises could significantly reduce pain, improve physical function and quality of life compared to the control group. It suggests that a modified Tai Chi exercises program was an effective treatment for elderly women with KOA. At the 3-month follow-up, our survey found that 65% of the Tai Chi group participants continued to do Tai Chi exercises after the end. Therefore, the intervention effect of modified Tai Chi exercise has been continued. The continuation of the intervention effect may be due to the modified Tai Chi being an easy-to-learn and very safe exercise. At the same time, the Tai Chi exercise effectively lessened pain in KOA patients, which stimulated the patient’s enthusiasm for continuing to learn and practice after the intervention. But at the 6-month follow-up, we found that the continuation of the effect of Tai Chi intervention decreased significantly because only 35% of participants continued to exercise. It indicated that long-term adherence to exercise might be key to sustaining intervention effects.

KOA patients generally have pain, stiffness, and restricted activities early. A recent meta-analysis showed that Tai Chi could be an alternative and effective treatment method to reduce pain and stiffness in KOA patients and improve physical function (Yan et al., 2013). The study results showed that at 12 weeks, the WOMAC pain in the Tai Chi group improved by 32% (24% higher than the control group); stiffness improved by 39% (35% higher than the control group), the physical function improved by 32% (a 27% improvement compared to the control group); this suggests a significant beneficial effect of Tai Chi on improving physical function because a 25% to 30% improvement in the WOMAC score is considered clinically meaningful.

Although the biological effect mechanism of Tai Chi exercises on improving KOA is still unclear, Tai Chi, as a slow and gentle exercise, improves the patients’ physical condition through many intermediate variables (Wang et al., 2010a). Tai Chi can trigger behavioral responses and change the sensitivity to pain by activating neuroendocrine and autonomic nerve functions, neurochemical and pain-relieving pathways (Irwin and Cole, 2011; Morgan et al., 2014). In addition, Tai Chi exercises can correct KOA patients with forceful posture and increase muscle strength to reduce pain. The pain-relieving effect of Tai Chi exercise may also be attributed to changes in the structure and activity of certain brain parts caused by Tai Chi exercises, such as the anterior cingulate gyrus and insular cortex (Gu et al., 2015). An investigative study showed that in people who received long-term Tai Chi exercise, increased cortical thickness in the inferior segment of the annular sulcus of the insular lobe was found, along with a decrease in the functional homogeneity of the left anterior cingulate cortex (Wei et al., 2013, 2014). Alterations in these brain regions may be related to Tai Chi-mediated pain modulation mechanisms, thereby explaining the pain relief effects of Tai Chi exercise in patients with KOA.

With respect to balance control, we found that Tai Chi exercises effectively improved the balance function compared to the control group. This was similar to the results of a previous trial of the effect of Tai Chi on postural stability in Parkinson’s patients (Li et al., 2012); these results support the benefit of Tai Chi exercise for improving balance function. During the practice, Tai Chi emphasizes the transfer of the body’s center of gravity by taking the trunk axis; the limbs move around the trunk in three-dimensional directions of front and rear, left and right, and up and down (Yu and Yang, 2012). It breaks the original static balance of the body, allowing the patient to continue to contract the muscles during practice and control the posture and stability (Gatts and Woollacott, 2006). The level of balance ability is also related to brain function, and long-term Tai Chi exercise is beneficial to improving brain function. Long-term Tai Chi exercise resulted in increased cortical thickness in the right precentral gyrus and improved homogeneity of the posterior central gyrus (Wei et al., 2013). It is well known that the precentral gyrus of the brain is the primary motor cortex responsible for coordinating and planning the voluntary movements of skeletal muscles, while the postcentral gyrus is the main sensory area. Thus, increased cortical thickness in the right precentral gyrus and improved homogeneity of the postcentral gyrus may provide the brain with more concise information on how to coordinate muscles for better balance control (Wei et al., 2014).

Compared with other chronic diseases, KOA has a greater impact on patients’ quality of life. Tai Chi Qigong showed its beneficial effects on KOA patients’ quality of life and physical function with no increased risks (Lee et al., 2009). As a potential form of postoperative rehabilitation exercise, Tai Chi exercise can effectively improve patients’ quality of life after total knee arthroplasty without increasing risks (Li et al., 2019). Our research confirmed that SF-36PCS and MCS improved significantly after 12 weeks of treatment. The effect of Tai Chi on quality of life appears to be associated with improved mental health, including reductions in stress, anxiety, depression, mood disorders, and increased self-confidence (Wang et al., 2010b). In addition, Tai Chi exercises use movement in conjunction with meditative attention, which helps combine with breathing for skeletal muscle stretching and relaxation, physical coordination, and high concentration (Li et al., 2001). From a behavioral immunology perspective, studies have found that the effectiveness of Tai Chi exercise in improving quality of life appears to be associated with significantly reduced sympathetic nervous system activity. Through salivary cortisol measurement, Tai Chi exercise can relieve psychological stress by producing regulatory T cell mediators transforming growth factor-beta and interleukin 10 under specific antigen stimulation, thereby improving the quality of life (Esch et al., 2007).

Previous research has shown that older adults with moderate sleep disorders can improve their sleep quality in six months using low-to-moderate-intensity Tai Chi exercises (Li et al., 2004). For instance, Lü et al. (2017) found that 24-weeks of Tai Chi exercise can improve total sleep time and sleep duration, and daytime dysfunction in elderly women with KOA. Our study also confirmed this finding, with an overall PSQI score improvement of 3.72 points (42% improvement) in the Tai Chi group compared to the control group. Although Tai Chi has been shown to help with sleep problems, no direct research reports prove that Tai Chi can improve sleep by altering brain structure or reducing sleep discomfort and insomnia. But studies have shown that meditation, an important part of Tai Chi training, can help patients improve sleep-related areas of the brain (Tao et al., 2017). Tai Chi meditation increases hippocampal volume and gray matter density in the orbitofrontal cortex; Tai Chi exercise has the potential to improve sleep quality by inducing similar changes in the brain (Luders et al., 2009). A recent study also showed a significant increase in resting functional connectivity between the brain’s bilateral hippocampus and prefrontal cortex after Tai Chi training (Tao et al., 2017). Although the changes in these brain regions caused by Tai Chi exercise are not directly similar to the brain changes observed in patients with sleep disorders, changes in related brain regions induced by Tai Chi exercise may be a possible mechanism for promoting sleep improvement. Therefore, Tai Chi exercise can be used as an important measure to improve the sleep quality of patients with knee OA.

The study found that psychological factors (such as mental health, depression, anxiety, etc.) may affect the response of KOA patients to exercise therapy and regulate pain symptoms by magnifying pain and fear avoidance (Gilpin et al., 2015; Lee et al., 2018). The present study showed that the anxiety and depression of patients in the Tai Chi group improved after 12 weeks, possibly because long-term Tai Chi exercises relieved tension and resulted in more frequent relaxation and calmness; greater happiness was achieved through mental concentration and physical exercise (Hartman et al., 2000). Tai Chi exercise intervention altered cortical thickness and functional connectivity in several mood-related brain regions; increased thickness of the insula’s right lower segment of the annular sulcus and improved functional specialization of the anterior cingulate cortex were observed in long-term Tai Chi exercisers (Wei et al., 2014). At the same time, resting-state functional connectivity between the ventromedial prefrontal cortex and the bilateral hippocampus was also increased (Tao et al., 2017). Increased functional connectivity between these brain regions may improve mood by associating current mood with previous events. Therefore, these brain changes induced by Tai Chi exercise may improve Tai Chi practitioners’ ability to deal with anxiety and depression, thereby alleviating the cause of the patient’s mood disorders.

Our study has several potential limitations, which should be resolved in future research. First, the results might be biased due to the small sample size. Therefore, future research should enroll more subjects. Second, the participants in this study consisted of older women with KOA, which limits the generalizability of the findings to other populations with KOA. Finally, a multi-subjective evaluation scale for the measure indicators in this study might have resulted in bias in understanding, leading to potential overestimation of the intervention effects.

## Conclusion

This study suggests that the modified Tai Chi exercise is effective in improving physical function and quality of life in elderly women with KOA. Furthermore, long-term adherence to Tai Chi exercise can be significantly beneficial to the continuity of the effect, which provides a basis for guiding patients to follow-up exercises. The modified Tai Chi exercise can be widely used in the treatment of clinical knee osteoarthritis.

## Data Availability Statement

The original contributions presented in the study are included in the article, further inquiries can be directed to the corresponding author/s.

## Ethics Statement

The studies involving human participants were reviewed and approved by Ethics Committee of Nanjing Sport Institute. The patients/participants provided their written informed consent to participate in this study.

## Author Contributions

JS, LW, KC, PX, AC, and XL designed the study. JS, XinW, BC, AD, and MS collected the data. JS and QL analyzed the data. TY, XiaW, AC, and XL provided advice related to the study. All authors contributed to the article and approved the submitted version.

## Conflict of Interest

The authors declare that the research was conducted in the absence of any commercial or financial relationships that could be construed as a potential conflict of interest.

## Publisher’s Note

All claims expressed in this article are solely those of the authors and do not necessarily represent those of their affiliated organizations, or those of the publisher, the editors and the reviewers. Any product that may be evaluated in this article, or claim that may be made by its manufacturer, is not guaranteed or endorsed by the publisher.
